# Current status of health technology reassessment of non-drug technologies: survey and key informant interviews

**DOI:** 10.1186/1478-4505-10-38

**Published:** 2012-12-14

**Authors:** Laura E Leggett, Gail Mackean, Tom W Noseworthy, Lloyd Sutherland, Fiona Clement

**Affiliations:** 1 Community Health Sciences and the Institute for Public Health, University of Calgary, 3280 Hospital Drive NW, Calgary, Alberta T2N 4N1, Canada; 2 Community Health Sciences, University of Calgary, 3280 Hospital Drive NW, Calgary, Alberta T2N 4N1, Canada

## Abstract

**Background:**

Health Technology Reassessment (HTR) is a structured, evidence-based assessment of the clinical, social, ethical and economic effects of a technology currently used in the health care system, to inform optimal use of that technology in comparison to its alternatives. Little is known about current international HTR practices. The objective of this research was to summarize experience-based information gathered from international experts on the development, initiation and implementation of a HTR program.

**Methods:**

A mixed methods approach, using a survey and in-depth interviews, was adopted. The survey covered 8 concepts: prioritization/identification of potentially obsolete technologies; program development; implementation; mitigation; program championing; stakeholder engagement; monitoring; and reinvestment. Members of Health Technology Assessment International (HTAi) and the International Network of Agencies for Health Technology Assessment (INAHTA) formed the sampling frame. Participation was solicited via email and the survey was administered online using SurveyMonkey. Survey results were analyzed using descriptive statistics. To gather more in-depth knowledge, semi-structured interviews were conducted among organizations with active HTR programs. Interview questions were developed using the same 8 concepts. The hour-long interviews were recorded, transcribed and analyzed using constant comparative analysis.

**Results:**

Ninety-five individuals responded to the survey: 49 were not discussing HTR, 21 were beginning to discuss HTR, nine were imminently developing a program, and 16 participants had programs and were completing reassessments. The survey results revealed that methods vary widely and that although HTR is a powerful tool, it is currently not being used to its full potential. Of the 16 with active programs, nine agreed to participate in follow-up interviews. Interview participants identified early and extensive stakeholder engagement as the most important factors for success. A lack of top-down support and financial and human resources are inhibiting program development.

**Discussion:**

HTR is in its infancy. Although HTRs are being conducted, there are no standardized approaches. However, much can be learned from current international work. Future work should focus on developing a comprehensive methodology, reporting the processes of reassessments and sharing successes and challenges in a common platform.

## Background

Health Technology Reassessment (HTR) has been garnering interest over the past decade as a method of improving quality of care and financial sustainability. HTR is a structured, evidence-based assessment of the clinical, economic, ethical, and social effects of a technology currently being used in the health care system, to inform optimal use of that technology in comparison to its alternatives [1]. There are a number of potential outcomes from HTR including the removal of funding from an ineffective technology, narrowing or broadening the scope of a technology’s use or sustaining existing funding [2]. As such, HTR is a tool that can improve public health, promote evidence-based decision-making and ensure optimal allocation of financial resources [3]. One of the goals of HTR is to identify areas of waste in a health care system by reassessing clinical value [2]. HTR is closely tied to Health Technology Assessment (HTA), yet takes place once the technology is already being used in clinical settings [4]. Technologies that are reassessed may have previously gone through the HTA process before they were introduced or may have never been assessed [2].

A recent systematic review revealed that there is only one published model for HTR [5]. The systematic review also found that a number of countries are actively completing health technology reassessments. However, there is little literature available on the practicality of successfully establishing a HTR program. The purpose of this study is to summarize experience-based information gathered through a survey and key informant interviews on the development, initiation, implementation and monitoring of a HTR program.

## Methods

### Data collection

A mixed methods approach, using a survey and in-depth interviews, was used to gather experiential knowledge. This approach was used in order to acquire an overview of the state of HTR activity internationally while gaining a deeper understanding of the mechanics and practicalities of established HTR programs.

A convenience sampling strategy was used. The sampling frame consisted of the organizational and individual members of Health Technology Assessment International (HTAi) and the organizational members of the International Network of Agencies for Health Technology Assessment (INAHTA); the two major international organizations of health technology assessment producers and users. A pre-notification email was sent to this sample seeking participation and requesting the name of a contact within their organization knowledgeable in HTR. Six days subsequently, an email containing the survey link was sent out. This link went out to the contacts who were identified as being involved in HTR and to the individuals who did not respond to the pre-notification email. The survey questions were developed to obtain information on eight broad areas that were defined a priori from a systematic review of the literature, as important facets of the HTR process: identification and prioritization of potentially obsolete technologies; program development; implementation; mitigation; program championing; stakeholder engagement; monitoring; and reinvestment. An online tool, SurveyMonkey, was used to conduct the survey from October 24th – November 7th, 2011.

The online survey was designed to stream participants into one of three sets of questions based on the organization’s stage of program development (Additional file 1: Appendix I). Respondents who indicated they had an active HTR program were also asked to participate in a voluntary follow-up interview. These interviews were conducted between November 22nd and December 14th, 2011. Interviews ranged in length from 30 to 90 minutes. A semi-structured interview guide was developed to guide these interviews. This guide evolved over the course of the interviews, as questions were refined to reflect what had been learned through the previous interview(s). Interviews were audio-taped and transcribed with the participants’ consent. Where no consent was given detailed notes were taken during the interview. Backup notes were taken for all interviews.

### Analysis

Basic descriptive statistics were used to summarize the survey findings. Frequencies and proportions were used to report the categorical variables. SurveyMonkey was used for all quantitative analysis.

Using constant comparative analysis, transcripts and notes were reviewed with the purpose of identifying key themes relative to the interview questions. Data management and analysis was facilitated through the use of mind-mapping software that supported the identification of key themes, and understanding the relationships between them.

## Results

### Survey results

**Figure 1:**
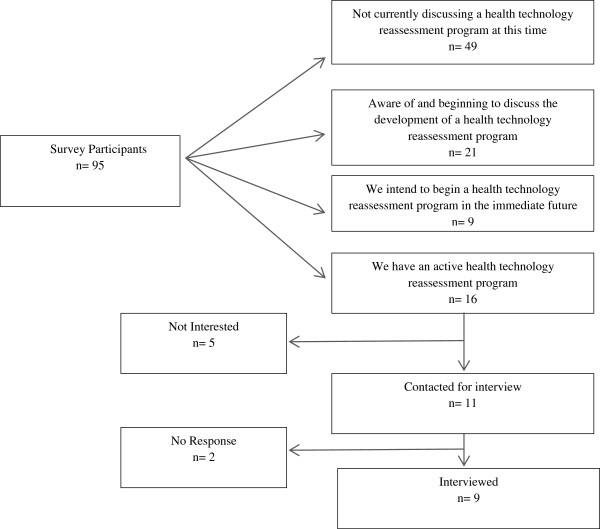
Flow chart of participant inclusion and exclusion.

The survey was sent to a total of 2,123 individuals; ninety-five responded (Figure 1). Of these, 49 (51.6%) did not have a HTR program and were not planning to develop one. Twenty-one (22.1%) noted that they were aware of and beginning to discuss the development of a HTR program. Nine (9.4%) responded that within the near future they would be developing a program; potential start-up dates for these programs ranged from 2011–2013. Sixteen participants (16.8%) identified that they had an established program and were currently reassessing health technologies.

Many countries were represented in the survey including Spain, Scotland, England, Norway, Australia, US, Argentina, Italy, France, Russia, Sweden, Thailand, Brazil, and Mexico. The majority (59.1%) of the survey respondents identified themselves as either researchers or senior managers. Nearly 34% responded that their role fit into an ‘other’ category, which included economists, professors, students, pharmacists and research fellows.

Nineteen of the 49 respondents who were not considering developing a HTR program cited that reassessment was not within their mandate. Seventeen of these nineteen respondents said that they had insufficient resources to develop the program, such as inadequate funding, time and qualified personnel, as the reason for not developing a HTR program.

**Figure 2:**
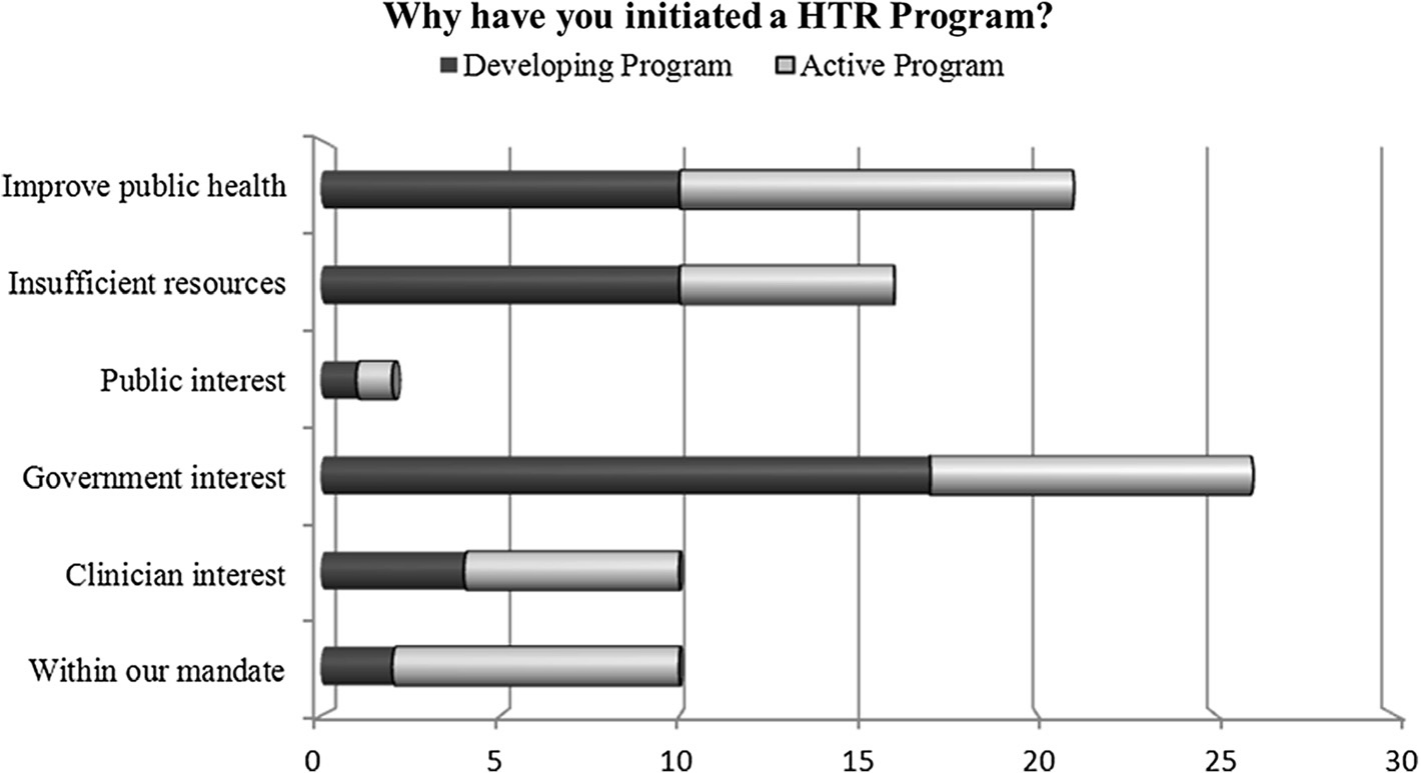
Survey question results from participants with established or developing programs.

**Figure 3:**
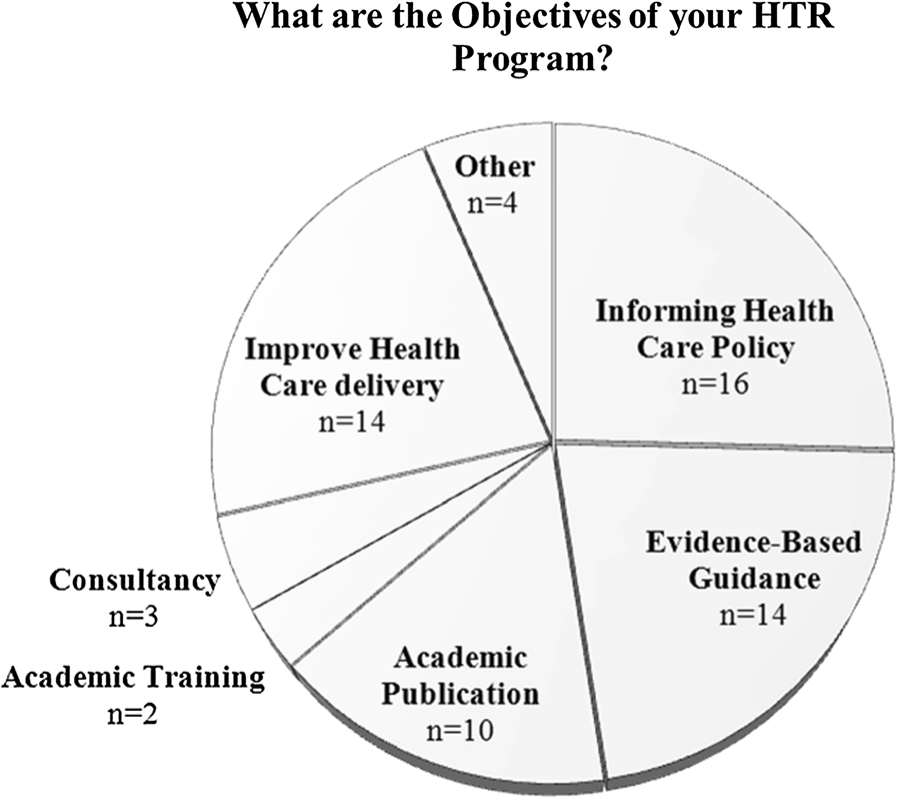
Survey question results from participants with established or developing programs.

Those with established or developing programs were asked why their organization decided to invest in the development of a HTR program (Figure 2). When the data from the active and developing programs were combined (n = 25), government interest was the most frequently cited reason for establishing a HTR program. Public interest was the least common reason for developing a program. Participants were additionally asked to identify the objectives of their program (Figure 3). Informing health policy was the most frequently cited objective (n = 15), followed by the creation of evidence-based guidance (n = 14) and improving health care delivery (n = 14).

In the survey, participants identified barriers they had encountered during development and implementation. Those still in the development stage responded that the top three barriers were: a lack of expertise in HTR (n = 10); political barriers (n = 9); and a lack of interest (n = 9). Those who have an active program cited political barriers (n = 10) as the most significant hurdle followed by a lack of expertise in the field (n = 6).

Nine of the sixteen individuals with active programs noted that their organization is responsible for mitigating barriers that arise during the HTR implementation process. Survey results suggest that most organizations use championing and stakeholder engagement as primary means of barrier mitigation. Common types of stakeholder engagement include: involving clinicians to increase buy-in; and having public representatives involved in the process to increase knowledge of the HTR process.

**Figure 4:**
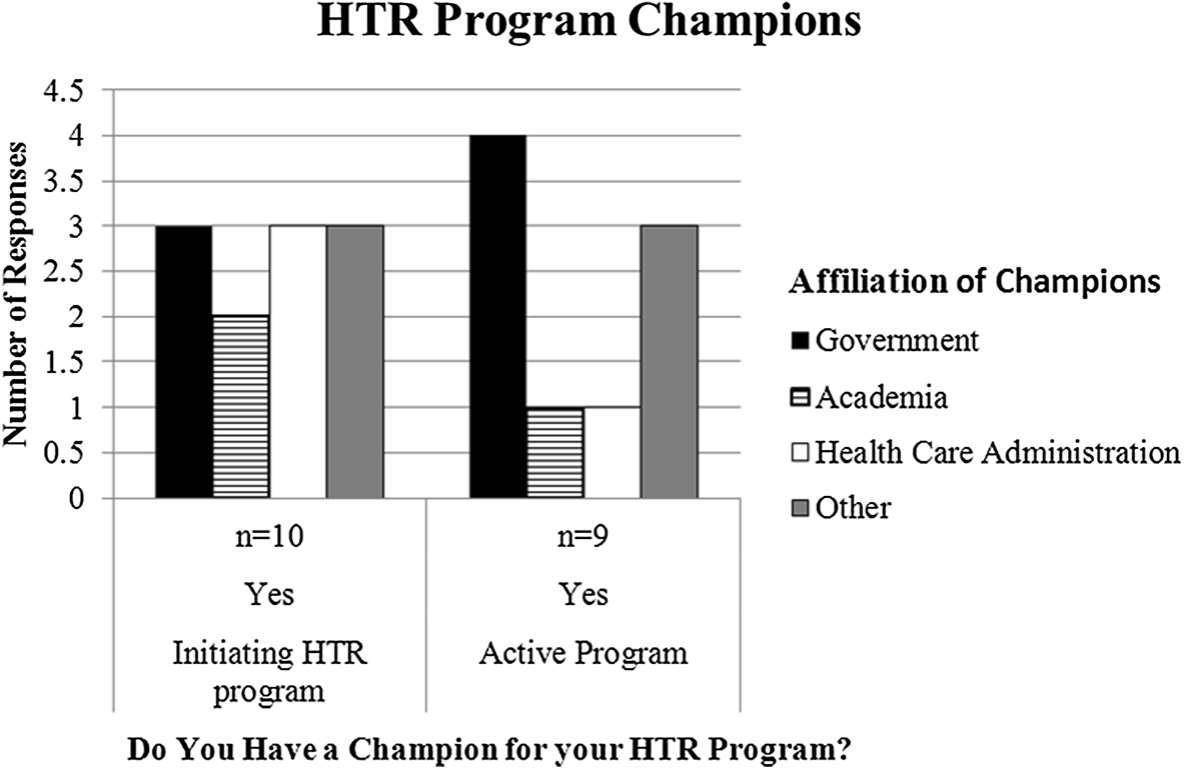
Survey question results from organizations actively completing reassessment projects.

Of those organizations actively doing health technology reassessments, 11 have a champion for the program (Figure 4). Of these eleven, nearly half responded that this champion was affiliated with government. Sixty percent (n = 14) of the individuals beginning to develop a program responded that they have a champion. In this case, the affiliation was evenly distributed between government, academia, and health care administration.

Participants with established or developing programs (n = 25) were asked how recommendations or decisions to reduce or remove funding for a technology would be implemented. Fifty-four percent of the organizations developing a program responded that they only have advisory capabilities; and therefore, the final reassessment decisions lie in the hands of an external decision-maker. Less than five percent responded that their organization had the ability to directly implement a HTR decision. Fifty percent of those who were part of an established HTR program said that they had advisory capabilities, whereas over 20% noted that they controlled funding decisions and therefore could implement their HTR decisions.

Participants who had established or developing programs, were also asked “If funds are removed from a technology that is reassessed, what happens to the resources that were allocated to it?” Four of the respondents with an established program noted that after disinvestment, funds are reallocated to a more efficacious technology; of those with a program in the development stage, eight noted that the funds were reallocated to a more efficacious technology. The survey results also revealed that many individuals involved in reassessment do not know what happens to funds that are liberated. Six organizations with developing programs and three with established programs were unsure of what would happen with liberated funds.

### Key informant interviews results

The sixteen survey respondents who indicated that their organization had an active reassessment program were invited to participate in a follow-up interview; nine indicated interest. These nine interview participants came from six jurisdictions: Australia, UK, Canada, Sweden, Costa Rica, and Italy; and consisted of policy decision-makers, researchers, senior managers, and government officials. As described previously, the purpose of these interviews was to obtain in-depth perspectives on the key questions asked in the survey.

With respect to reasons for initiating a HTR program, participants affiliated with academic settings generally noted that their HTR program was established with the goal of developing robust HTR models with rigorous economic evaluation components. Those affiliated with government tended to see HTR as a promising approach to improve fiscal sustainability in health care. At the health care delivery level, HTR was described as being a tool for advancing evidence-based practice while improving health care quality and safety. The increasing budgetary pressure facing governments and health care systems was also described as a key factor prompting the development of HTR programs.

Throughout the interviews, it became clear that there are many methods for completing reassessments, with little standardization internationally. Some interviewees explained that they did not have a separate HTR program, but rather had integrated HTR into their HTA work. One person working in a unit at the health service delivery level stated that: *“…lots of people do little projects that essentially have an element of disinvestment in them, but nobody is using that terminology.”* Organizations also conducted reassessments in varying degrees of depth. Some described taking a broader look at evidence-based practices (using both HTA and HTR), for example, reassessing all of the technologies from an entire clinical issue or area, whereas other organizations described reassessing one particular procedure or technology at a time.

The interview participants all encountered significant barriers while implementing their HTR programs. Some of the key barriers frequently cited included a poor evidence base, political push-back, a large investment of work and time for relatively small cost-savings, influence of industry, and difficulty communicating with a variety of audiences. Many methods for mitigating these barriers were proposed with the top two being stakeholder engagement and champion involvement.

Having a champion involved who can provide top-down support emerged from the interviews as a key strategy for developing and implementing a successful HTR program. This finding echoes the survey results presented earlier. During the interviews, one resarcher noted that HTR: "*…is potentially controversial and often, ideologically not well aligned with political interests, so having a political champion who is receptive…is important.”*

Interview participants described stakeholder engagement as necessary for success, noting that it was crucial to engage stakeholders promptly and continuously to be successful with HTR. As one senior government official commented: *“We take them on the journey with us.”* To this end, interview participants spoke at length about ways of engaging stakeholders; including the importance of presenting the reassessment findings in a way that is comprehensible and intuitive to a diverse health professional and public audience. Many approaches were highlighted in these conversations. Integrating reassessment findings, along with other kinds of evidence-based guidance, into care pathways was often brought up as a successful strategy, as this quote illustrates: “*The clinicians and even patients…found that the most interactive and the most easily understood [approach to presenting the guidance] was the pathway of care they needed to follow… Looking at all the appraisals and guidelines…they all need to feed into the same clinical area…[we have determined] that would be the way to best present our information.”*

Participants were also asked to describe the model, or framework, they used to complete their reassessments. Most frequently, the HTR models being used were developed by those delivering the HTR programs. It was regularly stressed that models were highly context-dependent (i.e., people develop models that will work in their unique context), meaning models developed elsewhere were difficult to adopt without modifications. None of the interviewees indicated that they had used a model developed elsewhere. Interview participants also emphasized that models and HTR processes tend to evolve over time based on successes and failures; so having a flexible model was imperative.

Although models and corresponding processes were developed to fit local context, many models do incorporate many of the same broad stages or steps. Most begin with a new or existing committee identifying and prioritizing potentially obsolete technologies for reassessment, while beginning to engage potential stakeholders and clinical experts. Prioritization is often based on some combination of utilization rates, ease of reassessment, mechanisms for change and potential cost savings. A review is then conducted on the selected technology and a recommendation or decision is put forth based on the collected evidence and input from stakeholders. The results of a reassessment are often made available through public websites, news releases and\or academic publications.

Since most final funding decisions are made at the governmental level, successful HTR implementation relies on processes outside the control of the organization conducting the review. One interviewee noted that approximately 50% of the time, the decision-making body accepted the recommendations coming from the review and advised the government to implement the decision. In this case, the government almost always acted on the Board’s advice. This rate of concordance reflects the political and social influences on decision-making.

Interview data suggests that reinvestment strategies are either lacking or unknown. This may be because monitoring processes are not in place and/or because reallocation decisions are beyond the scope of the organization’s mandate. Whether money saved can be reallocated back to the same clinical area toward a more effective technology is dependent on the type of savings and how budgets are allocated. In health care delivery organizations, for example, sometimes the savings occur in areas that are not aligned with a particular clinical department (e.g., decreased length-of-stay). One interview participant noted that it would be difficult to keep track of cost-savings: "*…trying to measure that without a health economist expertise is going to be really difficult. I think we may not be able to quantify the savings.”*

## Discussion

Results of this research indicate that many organizations realize the potential benefits of HTR and are interested in integrating it into their health care system. However, a lack of top-down support, push back from clinicians, financial and human resource limitations, and a lack of expertise in health re-assessment are inhibiting program development.

Those with developed programs offered sage advice for successful program development and implementation. It was often reiterated by interview participants that when launching a program, it is ideal to select technologies where the reassessment process is unlikely to be lengthy and will result in significant fund liberation. This will increase the likelihood that public understanding of HTR will be improved and get relevant stakeholders on board.

The importance of learning from successes and failures, and having the flexibility to adjust models and processes accordingly was frequently highlighted. Similarly, it was continually reiterated that communication with stakeholders is critical to the success of a HTR program. Stakeholders must be engaged early, continuously and as extensively as resources allow. With implementation, it is important to have people and processes in place to move a decision into practice and policy.

There is no commonly accepted model for reassessing health technologies and very little agreement on specific methods amongst those completing HTRs. Although interview participants revealed that models must be very context-dependent, having even a broad outline of steps and stages based on effective HTR programs may help develop more successful programs.

Such a model must include reinvestment and monitoring; two elements infrequently incorporated into current models. In the development of these stages, international collaboration and perspectives would increase the applicability of such a model. There are a few experts who possess a wealth of experience-based knowledge on the subject. Since there are few people who have in depth knowledge of this field, many people indicated that not having expert knowledge within their organization was preventing them from developing a HTR program. For this field to develop to its full potential, an emphasis on communication and collaboration between interested organizations is essential. Focusing on using collaboration and knowledge-sharing as a means to move the HTR agenda forward has been emphasized in current literature [6-8].

This study has limitations. The survey response rate was low; as this is a new and small field, this was anticipated. The sampling frame was intentionally broad and over-inclusive to ensure that all potential respondents were captured. The number of key-informants was small. However, many of the same themes were repeated across the interviews, indicating that we were close to achieving saturation in the thematic areas reported here.

## Conclusions

HTR has the potential to improve the quality of care patients receive, promote evidence-based practice and improve health care sustainability. It is a powerful tool that is not being used to its full potential internationally. However, it is currently limited by an under-developed theoretical base, a small field of experts and limited reporting of case studies in the literature. Future work should focus on advancing the theoretical framework for HTR drawing upon developed theories in knowledge translation and implementation science. In addition, case reports of HTR applications that include a strong monitoring and evaluation component should be reported in the scientific literature. This will contribute to the development of a body of knowledge from practice that has great potential to improve our understanding of what works and what does not work in which contexts and why, that those embarking upon HTR could draw upon.

## Abbreviations

HTR: Health technology reassessment; HTA: Health technology assessment; HTAi: Health technology assessment international; INAHTA: International network of agencies for health technology assessment.

## Competing interests

None of the authors have competing interests to disclose.

## Authors’ contributions

Design of the study LL, TN, LS, GM, FC; collection of data LL, GM, TN FC; management of data LL, GM, analysis of data LL, GM, FC; interpretation of the data LL, GM, FC; preparation of manuscript LL, FC; review of manuscript LL, GM, TN, LS, FC; approval of manuscript LL, GM, TN, LS, FC. All authors read and approved the final manuscript.

## Funding/Support

Alberta Health.

## Supplementary Material

Additional file 1: Appendix 1 Survey Questions. Click here for file

## References

[B1] Noseworthy T Clement FM Health technology reassessment: scope, methodology and language Int J Technol Assess Health Care 2012 28 3 201 202 10.1017/S0266462312000359 22980695

[B2] Joshi NP Stahnish FW Noseworth TW Reassessment of Health Technologies: Obsolescence and Waste 2009 http://www.cadth.ca/media/pdf/494_Reassessment_of_HT_Obsolescence_and_Waste_tr_e.pdf

[B3] Elshaug AG Hiller JE Moss JR Exploring policy-makers perspectives on disinvestment from ineffective healthcare practices Int J Technol Assess Health Care 2008 24 1 9 18218163 10.1017/S0266462307080014

[B4] Center for Health Economics Research and Evaluation Reducing the Use of Ineffective Health Care Interventions 2010 http://www.chere.uts.edu.au/pdf/wp2010_5.pdf

[B5] Leggett LE Noseworthy T Zarrabi M Lorenzetti D Sutherland L Clement F Health technology reassessment of non-drug technologies: current practices Int J Technol Assess Health Care 2012 28 3 220 227 10.1017/S0266462312000438 22980697

[B6] Elshaug AG Hiller JE Tunis SR Moss JR Challenges in Australian policy processes for disinvestment from existing, ineffective health care practices Aust New Zealand Health Policy 2007 4 23 10.1186/1743-8462-4-23 17973993 PMC2174492

[B7] Cooper C Starkey K Disinvestment in health care BMJ 2010 340 c1413 http://www.bmj.com/content/340/bmj.c1413.extract 10.1136/bmj.c1413

[B8] Metropolitan Health and Aged Care Services Division Future Directions for Health Technology Uptake, Diffusion and Disinvestment in Victorian Public Health Services: Department of Human Services Workship Discussion Paper 2007 http://www.health.vic.gov.au/newtech/documents/new-tech-workshop-discussion.pdf

