# Supplementary material for: Current status of health technology reassessment of non-drug technologies: survey and key informant interviews
Source: Health Res Policy Syst. 2012 Dec 14;10:38. doi: 10.1186/1478-4505-10-38 (PMC3542085; doi:10.1186/1478-4505-10-38)
Supplement: Additional file 1: Appendix 1 — Survey Questions. [file 1478-4505-10-38-S1.doc]

# APPENDIX 1: Online Survey Questions

1. Name of Organization/Institution: (text box)

|  |
| --- |

2. What is your primary role within the organization?

- Researcher
- Director
- Vice President
- President/CEO
- Health Care Provider
- Public Relations
- Program Adviser
- Supervisor
- Other (please specify)

|  |
| --- |

3. Which of the following statements best describes your organization?

- We are not currently discussing a HTR program at this time
- We are aware of and beginning to discuss the development of a HTR program
- We intend to begin a HTR program in the immediate future
- We have an active HTR program

**Stream 1: We are not currently discussing a HTR program at this time**

4. Please indicate why you are not currently discussing HTR initiatives at this time (select all that apply):

- It is not within our group's mandate
- It is another group's responsibility
- No interest from government
- No interest from clinicians
- No interest from leadership
- Insufficient financial resources to start initiative
- Insufficient human resources to start initiative
- Insufficient expertise to start initiative
- Sufficient resources (HTR isn't necessary)
- Other (please specify)

5. Thank you so much for completing this survey. Feel free to contact us by email (htrr@ucalgary.ca) should you have any questions. If you have any further information or suggestions relevant to HTR, we would appreciate your comments below. (Text Box)

**Stream 2: We intend to begin a HTR program in the immediate future/we intend to begin a HTR program in the immediate future**

4. Please indicate the tentative start-up date of your HTR initiative (e.g. April 2012): (text box)

5. Please indicate why you are initiating a HTR initiative (select all that apply):

- It is within our mandate to do so
- Clinicians have expressed an interest in a HTR program
- The government is expressing an interest in HTR
- The public has expressed an interest in a HTR program
- Currently, our health care system has insufficient resources to be financially sustainable
- It is a method of improving public health
- Other (please specify)

6. How important is it to your organization to establish a HTR program?

- Very important
- Important
- Moderately important
- Of little importance
- Unimportant

7. Do you have a champion (a person or group who promotes and advocates) for your HTR program?

- Yes
- No

8. If yes, at what structural level does your champion reside?

- Government
- University/academia/research
- Clinical
- Healthcare administration
- Other (please specify)

9. Do you plan to model your HTR Program after an existing program?

- Yes
- No

10. If yes, please identify the HTR program you intend to use as your model: (text box)

11. What are the objectives of your HTR program? (Select all that apply)

- Informing healthcare policy
- Evidence-based guidance
- Academic publication
- Academic training
- Consultancy
- Improve health care delivery to citizens
- Other (please specify)

12. Once your organization decides to remove or reduce the funding allocated to a technology, how will that decision be put into practice (select all that apply)?

- Our organization makes recommendations to decision-makers
- Our organization makes funding decisions
- We produce clinical guidelines
- We produce technical guidelines
- Other (please specify)

13. Once funding for a technology is removed or reduced, what happens to the funds that were allocated to it?

- They will be reinvested in a more efficacious technology
- They will be reinvested in other health initiatives
- They will be reinvested in a non-health related initiative
- They will not be reinvested
- Unsure
- Other (please specify)

14. Which of the following barriers have you encountered during the development of your HTR program? (Select all that apply)

- Lack of interest
- Financial
- Expertise
- Scientific
- Technology
- Ethical
- Political
- No barriers
- Other

15. How have you mitigated these barriers? (Text box)

16. Thank you so much for completing this survey. Feel free to contact us by email (htrr@ucalgary.ca) should you have any questions. If you have any further information or suggestions relevant to HTR, we would appreciate your comments below. (Text box)

**Stream 3: We have an active health technology HTR program**

4. How long has your program been active? (Text box)

5. Please indicate why your organization initiated a HTR initiative (Select all that apply):

| - It is within our mandate to do so |
| --- |
| - Clinicians expressed an interest in a HTR program - The government expressed an interest in HTR - The public expressed an interest in a HTR program - Our health care system had insufficient resources to be financially sustainable - Other (please specify) |
|  |

6. Once your organization decides to disinvest a technology, how is that put into practice? (Select all that apply)

| - Our organization makes recommendations to decision-makers |
| --- |
| - Our organization makes funding decisions |
| - We produce clinical guidelines - We produce technical guidelines - We have not yet completed a reassessment - Other |

7. Is your current HTR program modeled after an existing program?

| - Yes - No |
| --- |

8. Please identify which HTR program you used as your model: (Text box)

9. Do you have a champion (a person or group who promotes and advocates) for your HTR program?

| - Yes |
| --- |
| - No |
|  |

10. At what structural level does your champion reside?

| - Government |
| --- |
| - University/academia/research |
| - Clinical - Health care administration - Other (please specify) |

11. How important is your HTR program to your institution/organization?

- Very important

| - Important |
| --- |
| - Moderately important |
| - Of little importance - Unimportant |

12. Which of the following barriers did you encounter while developing your HTR program? (Please select all that apply)

- Lack of interest
- Financial

| - Expertise - Scientific |
| --- |
| - Ethical |
| - Political |
| - Other (please specify) |

13. How were these barriers mitigated? (Text box)

14. Is your group involved with identifying potentially obsolete technologies?

| - Yes |
| --- |
| - No |

15. What sources of information do you use to identify potentially obsolete technologies? (Text box)

16. What criteria does your group use to determine what technology should be prioritized for HTR? (Text box)

|  |
| --- |

17. Has your health care system encountered barriers when disinvesting a technology?

| - Yes |
| --- |
| - No |

18. Please identify the barriers encountered: (Text box)

|  |
| --- |

19. Was your group involved in mitigating these barriers?

| - Yes |
| --- |
| - No |

20. How were these barriers mitigated? (Text box)

21. How would you rate the success of your HTR program?

- Very successful

| - Slightly successful - Neutral - Slightly unsuccessful - Very unsuccessful |
| --- |
|  |

22. How do you measure the success of your program? (Please select all that apply)

| - Number of completed assessments |
| --- |
| - Number of published assessments |
| - Changes in practices and procedures |
| - Assessments that have resulted in policy change |
| - Number of students trained |
| - Changes to clinical guidelines |
| - Changes to technical guidelines |
| - Reallocation of funding - Other (please specify) |

23. Please provide the number of reassessments your organization has completed per year: (Text box)

24. Have you made your reassessments available to the public?

| - Yes |
| --- |
| - No |
|  |

25. In what form has the information been made available to the public? (Please select all that apply)

| - Website - News media |
| --- |
| - Newsletter - Books - Reports |
| - Scientific publications - Information seminars/town hall meetings |
| - Other (please specify) |

26. Once a technology is disinvested, what happens to the funds that were allocated to it?

| - Funds are reinvested in a more efficacious technology - Funds are reinvested in other health initiatives - Funds are reinvested in a non-health related initiative - Funds are not reinvested (they are saved) - Unsure - Other (please specify) |
| --- |

27. If possible, please provide a link to websites, publications, news stories etc. developed for the purpose of public awareness of your technology reassessments: (Text Box)

|  |
| --- |

28. We are very interested in learning more about your organization's HTR program. At some point within the next month, would you be open to having a 15 minute conversation with us about your program?

| - Yes |
| --- |
| - No |

29. If yes, we look forward to speaking with you. Please provide your name, a phone number we can reach you at and your availability: (Text box)

|  |
| --- |

30. Thank you so much for completing this survey. Feel free to contact us by email (htrr@ucalgary.ca) should you have any questions. If you have any further information or suggestions relevant to HTR, we would appreciate your comments below. (Text box)
